# Strong site fidelity, residency and local behaviour of Atlantic cod (*Gadus morhua*) at two types of artificial reefs in an offshore wind farm

**DOI:** 10.1098/rsos.240339

**Published:** 2024-07-10

**Authors:** B. J. P. Berges, I. van der Knaap, O. A. van Keeken, J. Reubens, H. V. Winter

**Affiliations:** ^1^ Wageningen Marine Research, Yerseke, The Netherlands; ^2^ van Hall-Larenstein University of Applied Sciences, Leeuwarden, The Netherlands; ^3^ Flanders Marine Institute (VLIZ), Oostende, Belgium; ^4^ Aquaculture & Fisheries Group, Wageningen University, Wageningen, The Netherlands

**Keywords:** animal telemetry, offshore wind farm, artificial reefs

## Abstract

Globally, biogenic temperate reefs are among the most threatened habitats. In the North Sea in particular, large shellfish reefs were lost owing to fishing activities in the 1900s. The impact of offshore wind farms (OWFs) on marine wildlife is extensive, and it offers the possibility to reintroduce new hard substrate habitats that are protected from fisheries at a large scale. In addition to the submerged structures of OWFs, marine hard substrate habitat can be further enhanced by providing extra artificial reefs. In an operational OWF along the Dutch coast, four artificial reefs (two with a scour bed and two without) were deployed in the vicinity of a wind turbine. Acoustic telemetry was used to monitor the fine-scale movement of 64 Atlantic cod (*Gadus morhua*). The monitoring ran from July 2021 to January 2023. Detailed information on behaviour, area utilization and attraction to the structures was determined. Results showed strong attraction (high site fidelity and residency) to the artificial reef, with no significant difference between the two tested types of reefs, and only a few individuals staying over winter. Cod spent a large proportion of their time hiding in the artificial reefs, suggesting that adding pipes for shelter has a beneficiary effect.

## 1. Introduction

Biogenic temperate reefs have been among the most threatened habitats worldwide owing to bottom trawling and dredging [1]. In the North Sea, all large oyster reefs (over 25 000 km^2^) were lost in the early 1900s [2,3]. These biogenic reefs provided a hard substrate for many associated life forms. The recent and future rapid development of large-scale offshore wind farms (OWFs) in the North Sea [4] provides new hard substrate habitats, e.g. submerged vertical structures and rocky scour beds, that can act as artificial reefs for a diverse epifauna community [5]. This constitutes an opportunity despite that only a small fraction of OWFs will consist of hard substrate, i.e. below historical areas with oyster beds. In some countries, fish communities can also be protected with the exclusion of bottom trawling in OWF areas. Overall, OWFs provide opportunities to additionally deploy designated artificial reefs to further support marine life associated with hard substrate habitats [6].

The impact of OWFs on marine wildlife is a mixture of positive and negative effects. Negative effects of OWFs can occur during the construction and operation phase, for example, disturbances to the surrounding marine environment introduced by noise, electromagnetic fields around cables and degradation of habitats [7]. Conversely, positive effects for marine wildlife include species aggregation and protection from fishery activities (sanctuary function). Multiple studies showed that the submerged hard substrate structures in OWFs can act as artificial reefs attracting benthopelagic fish species [8–11]. The underlying mechanisms for this attraction can be various, ranging from feeding on local prey associated with the hard substrate habitats, seeking shelter against predation or reducing energy costs in high current environments, and depending on species and life stage [12]. To what degree fish species that are associated with artificial reefs benefit from these newly created habitats on a population level depends on factors such as life traits and infrastructure layout (i.e. type of structure). Whether fish are merely attracted (i.e. change in spatial distribution with no net population benefit) or whether local production is affected with a positive net population effect (because of increased growth or survival rate) is still subject to debate for many species and known as the ‘attraction–production issue’ [13,14]. If attraction to these artificial reefs is high and associated with increased mortality or reduced growth, like what has been shown for tuna at fish-attracting devices (FADs) in the open ocean [15], they can even act as ecological traps with a negative net population effect [16].

For Atlantic cod (*Gadus morhua*), these factors in relation to hard substrate habitats in OWFs are relatively well studied in the southern North Sea [9,12,17]. In addition, for Atlantic cod, strong attraction to OWF structures was observed, with higher catch per unit of effort (CPUE) compared with shipwrecks or soft sediment habitats [18] and high site fidelity and residency as determined with acoustic telemetry [17,19]. Feeding occurs on local epifauna on OWF structures as revealed from diet studies [20] and combined diet–stable isotope studies [21]. In OWFs, the abundance of Atlantic cod is higher during summer [9,12], followed by migration in winter [12], though presence in winter has been observed [9,22]. Across summer and winter seasons, fish at various maturity stages were observed, including the potential for spawning [22]. Furthermore, stomach content analysis revealed that turbines and scour protections in OWFs are associated with long residency of cod and provide favourable conditions with a suitable and diverse prey field [22]. OWFs in the southern North Sea, especially those where fishing is excluded, have the potential for positive net population effects, even though proof for an increase in the production at larger scales is not available at present [12]. One of the reasons for this might be the relatively small area of hard substrate habitats provided by current OWFs. OWFs could offer opportunities for climate change mitigation and species conservation [22], especially in the context of the decline of the southern North Sea cod stock [23].

With increasing awareness towards ecosystem protection, OWF operators are triggered to invest in finding solutions to (i) prevent or mitigate detrimental effects and (ii) define and develop ecological beneficiary measures, e.g. by enhancing marine habitats within OWFs with additional artificial reefs. Owing to the novelty of such a measure, short- and long-term effects (i.e. potential change in behaviour around the artificial reefs) need to be carefully assessed.

For habitat enhancement, two types of artificial reef structures (i.e. consisting of concrete pipes of various diameters with or without a scour bed) were placed in a Dutch OWF. It has been shown that scour protection is an important feature of artificial reef structures for the habitat suitability of Atlantic cod [6,24]. In the southern North Sea, larger catch rates were observed around turbines with rock protection [22,24]. To test the effect of the two reef types (with and without scour bed) on fish, a field experiment was carried out around these artificial reefs (in duplo) in addition to the already present submersed structures around monopiles in the OWF. Atlantic cod was chosen as a test species, based on the strong knowledge base on cod behaviour in OWFs, its suitability for using telemetric methods to track fine-scale individual behaviour [17] and its importance for fisheries. The objective of this study was to assess the use of the artificial reefs by cod by (i) investigating changes in site residency and fidelity, (ii) investigating behavioural patterns, and (iii) testing for differences in site residency, fidelity and behaviour between the two types of artificial reefs.

## 2. Material and methods

### 2.1. Study area and acoustic telemetry network

**Figure 1.**
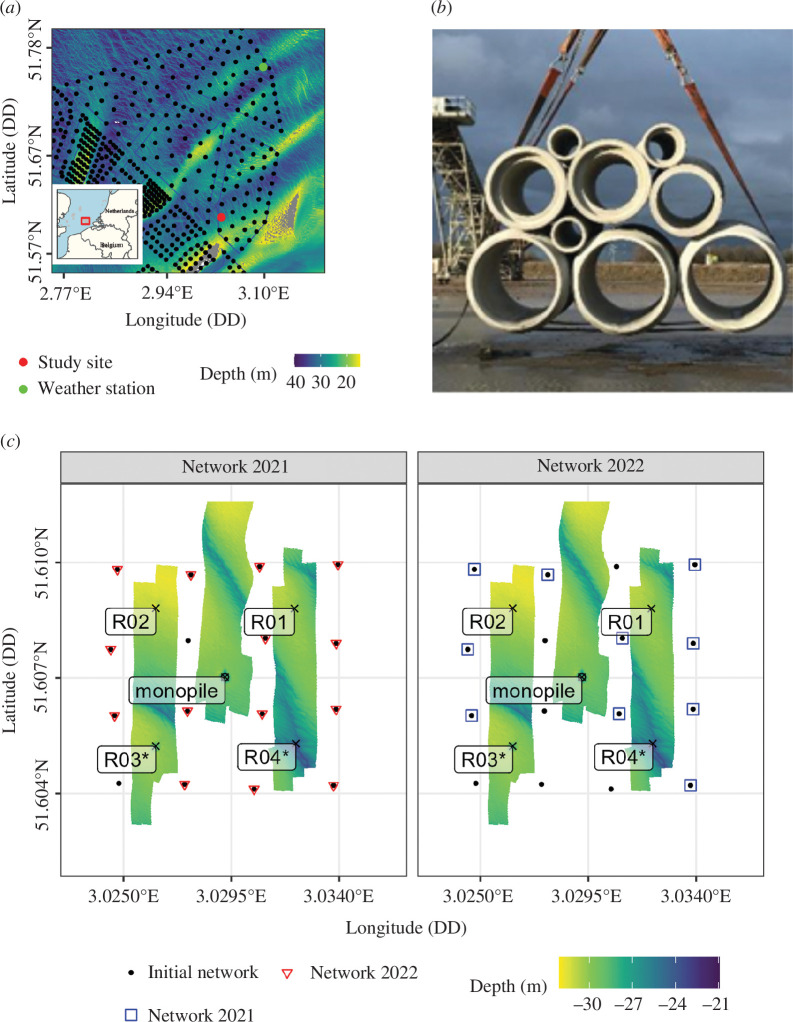
Study set-up. (*a*) Overview of the study area (red rectangle). The monitoring took place around a single monopile in the Borssele OWF in Dutch waters. (*b*) Photograph of the type of concrete pipes deployed on the seabed as artificial reefs. The arrangement of pipes is given in table 1. (*c*) Acoustic telemetry network and surrounding structures (R01–R04 and monopile L08). Because of the loss of equipment, the two datasets considered in this study had altered coverage (red downward triangles for the 2021 dataset and open blue rectangles for the 2022 dataset) relative to the initial deployment (solid black circles). Scour protection was installed around the monopile and the southernmost artificial reefs (R03–R04, labelled with an asterisk). The rock protection consisted of a rock pad with a top-footprint radius of 12.5–14.5 m and a layer thickness of 0.7–1.2 m.

The study took place between 25 February 2021 and 30 January 2023 in the Borssele II wind farm zone, located along the Dutch coast (figure 1*a*). The study site was limited to an area with a depth of approximately 30 m with a single monopile in the middle and four artificial reefs that were deployed around it in July 2020 (figure 1*a,b*). The four reefs were placed at approximately 280 m from the monopile in NW, NE, SE and NW directions between 1 and 17 July (figure 1*c*, R01–R04). Each reef consisted of an assembly of concrete pipes deployed on the seabed, with an arrangement varying 0.5–1.5 m in diameter (table 1). The same scour protection was installed around the monopile and underlying the two southernmost artificial reefs. The rock protection consisted of a rock pad with a top-footprint radius of 12.5–14.5 m and a layer thickness of 0.7–1.2 m.

**Table 1.** Reef information and animal release at site.

structure	number of pipes 0.5 m	number of pipes 1.0 m	number of pipes 1.5 m	scour protection	batch 2021 number of fish released	batch 2022 number of fish released
R01	21	12	12		12	4
R02	21	12	12		8	7
R03	21	12	12	yes	13	3
R04	21	12	12	yes	12	5
L08	–	–		yes	0	0

To track the individual movement of cod in the study area, acoustic telemetry was used. A total of 16 receivers (InnovaSea VR2AR) mounted on 1.5 m tall stainless steel tripod frames (80 kg) [25] were deployed in a 4 × 4 grid (200–250 m distance between receivers) allowing for fine-scale triangulation of fish positions (figure 1*c*). Each acoustic receiver was positioned vertically. The average tilt angle was 7.9° (s.d. 6.3°). Receivers were deployed on 25 February 2021, retrieved for a read-out, redeployed on 13 April 2022 and retrieved again on 30 January 2023. The positions of the receivers were logged using a handheld global positioning system (GPS) and additional position corrections were made after receiver retrieval based on position triangulation of the internal transmitter. During the retrieval operation on 13 April 2022, 14 receivers were retrieved and 13 were redeployed, and of these, 11 were retrieved on 30 January 2023. Owing to the loss of receivers, coverage was particularly reduced in 2022 in the southwestern corner where reef R03 is located, with three receivers lost out of four initially deployed.

### 2.2. Fish tagging

Cod were tagged with InnovaSea V13AP coded transmitters with pressure and acceleration sensors (length 39 mm, diameter 13 mm, weight 11 g in air, weight 5.5 g in water, estimated battery life 278 days, 69 kHz), which allowed for detailed three-dimensional tracking of individual movements in the study area [17]. The transmitters were set to a random delay of 50–100 s between signals and alternating pressure and body acceleration measurements. Acceleration was measured as the cumulative means over 37 s in the three-dimensional vector dynamic body acceleration (VeDBA) [17].

In total, 64 cod were caught at the four artificial reefs, tagged and released at the reef site where they were caught (table 1); 45 (total length (TL) 32–56 cm, average 39.3 cm) on 10–12 July 2021; 19 (TL 32–58 cm, average 36.9 cm) on 15–20 May 2022 (electronic supplementary material, figure S1 and table S1). Tags did not exceed 2% of fish body weight [26] and only cod greater than 32 cm were tagged [27]. For both tagging rounds, fishing at the monopile was undertaken but unsuccessful, partly because of the difficulty to lower the bait without catching pelagic species. Cod were reeled in at low speed to minimize barotrauma [28] and only cod without any signs of barotrauma were tagged. Fish were measured to the nearest centimetre below and anaesthetized using a 0.4 ml l^−1^ 2-phenoxyethanol solution. Tags were surgically implanted in the body cavity by making a mid-ventral incision of approximately 2 cm in the posterior quarter of the body cavity that was closed with two sutures (absorbable, braided Vicryl 3/0, FS2 needle). A floy-tag was inserted below the dorsal fin, so fish could be reported when recaptured by fishermen. The surgery lasted 5 min at maximum. All fish were transferred to a recovery tank and observed until ‘normal’ swimming behaviour reappeared and consequently released at their catch location (table 1).

### 2.3. Fine-scale positioning

Detections at all receivers were processed using the Fathom Position web platform by InnovaSea.^1^ Using the reference acoustic emission from each acoustic receiver, the clock drift of a receiver was corrected for using linear regressions over the time difference between different receivers [29]. This correction allowed the time synchronization of detections across the receiver network and was an essential step to further compute accurate positioning. From the synchronized detections, two-dimensional locations were computed using the time difference of arrival (TDOA) [30]. To calculate a two-dimensional position, simultaneous detection by at least three receivers was required.

Alongside positioning using TDOA, an indicator of accuracy for each position was computed [29], so-called horizontal position error (HPE). The HPE is a dimensionless quantity calculated through multiple receiver combinations. The profile of HPE is specific to each acoustic network set-up, i.e. not comparable to other studies. Similar to [17,31,32], the HPE associated with each cod position was used to filter out deviates. Here, the highest 2% HPE quantile was filtered out. In addition, detections associated with temporal and spatial trends indicating tag loss or death were removed.

### 2.4. Data analysis

#### 2.4.1. Spatial use

In order to investigate the spatial utilization of the study area and determine home-range size, utilization distributions (UD95) were computed using kernel densities with the ‘adehabitatHR’ R package [33]. When fitting kernel densities to estimate home range, the most important parameter is the smoothing factor as it underpins the fitting of the kernel density. A fixed value of *h* = 6 was chosen over a parametric estimation because of the large number of cases (number of individuals and number of days for each) and the need to standardize the calculations over the different individuals. As for the magnitude of smoothing, based on examining the daily spatial distribution of fish positions, a value that best captured point densities was chosen, optimizing the degree of under- and over-smoothing [34–36].

Home ranges were fitted daily for each individual over day and night periods (electronic supplementary material, figure S2, for examples). Individual home ranges were defined as the smallest area containing 95% of the UD (UD95). Home ranges were further filtered based on the following criteria: (i) the polygon should be derived from at least six positions, (ii) the time extent of the positions within the polygon should be greater than 2 h. Each home-range polygon was further associated with structures in the study area (reef, turbine, acoustic receivers). To associate each polygon to a structure, it was first envisioned to have a condition on the containment of the position of the structure in the home-range polygon. However, it was not satisfactory in few cases where the home-range polygon was in the vicinity of the structure but did not contain it. When deploying each receiver, GPS positions were taken from the water surface, which introduces an uncertainty on the position of each receiver on the seabed. While the location of each receiver was calibrated relative to a single receiver, an offset of a few metres might have persisted in the process. Consequently, the association between home-range polygons and structures was done based on the distance from the structure to the centroid of the home-range polygon. The association was made with polygon centroids at a distance less than 30 m from a structure. Site fidelity was derived for each home-range polygon and represents residency over a small time scale (i.e. per animal, day and day/night periods). Over a given period (per animal, day and day/night period), the total number of existing positions is N and a subset $N_{p}$ are contained within a home-range polygon. The fidelity to this home-range polygon is derived as: $F=N/N_{\text{p}}$ .

#### 2.4.2. Inferring hiding in artificial structures

The four artificial reefs consisted of concrete pipes which provided shelter to the animals. When entering the pipes, the acoustic emissions of the tags are expected to be strongly reduced, hampering the ability to derive fine-scale positioning. To determine the time at which the animals hide in the pipes, a non-spatial hidden Markov model (HMM) was used using the ‘momentuHMM’ R package [37]. HMM is a widely used modelling framework for inferring latent animal behaviours (hidden states) given a sequence of observations (data streams) configured by probability distributions [38]. The input data were binned per 10 min based on all detections (with and without successful positioning). The HMM was configured with three data streams: (i) the mean distance to the closest reef, (ii) the number of unique receiver IDs, and (iii) a binary on the presence of at least one successfully inferred TDOA position. For data stream (i), it is important to note that the distance to the closest reef could only be computed when TDOA was successful. For intervals without successful TDOA positioning, the distance to the closest reef was interpolated based on the closest values. The distance to the closest reef and the number of receiver data streams were modelled using a Gamma distribution parametrized with mean and standard deviation. The binary on TDOA positioning presence was modelled with a Bernoulli distribution, parametrized with the probability of occurrence. Four different states for the HMM were defined based on the three data streams: (i) at reef without TDOA positioning (i.e. hiding within the concrete pipes, HS1), (ii) at reef with TDOA positioning (HS2), (iii) not at reef with TDOA positioning (HS3), and (iv) not at reef without TDOA positioning (HS4). Using the HMM, the sequence of states was decoded using the Viterbi algorithm [37] and could be associated with each 10 min interval. Using these decoded states, the proportion of hiding in the structures (HS1) was computed per day (based on 10 min bins) for each animal. The proportion of hiding was further modelled using a general additive model (‘mgcv’ R package [39]). Model covariates were individual fish ID (smooth with random effect, *k* = 45), artificial reef location (as categorical), mean daily current speed (m s^−1^, as smooth, *k* = 9), day of year (as smooth, *k* = 9) and daily number of individual ID detected (as smooth, *k* = 5). The last was particularly important because the presence of numerous tagged animals may hamper the effective detection of the acoustic tags owing to the collision of signals and may inflate the estimation of the hiding in the concrete pipes of the artificial structures (HS1).

#### 2.4.3. Behaviour analysis

In order to investigate behavioural patterns around the artificial reefs, a spatial HMM was fitted to the calculated animal two-dimensional positions using the ‘momentuHMM’ R package [37]. The HMM was configured similarly to van der Knaap *et al*. [17] with step length (distance between two consecutive positions) and fish body acceleration (VeDBA) as data streams to estimate fish behavioural states (BS). Both data streams were modelled using a Gamma distribution, parametrized with mean and standard deviation (s.d.). The HMM was based on positions grouped per 10 min. The number of BS was based on acoustic telemetry studies on Atlantic cod [17,40]: locally inactive (BS1), locally active (BS2) and transit (BS3). The parameters of the data streams as estimated by van der Knaap *et al*. [17] were used as initial conditions for the model. The HMM model was fitted using an individual random effect by adding the individual fish ID as a covariate to transition state probabilities. Additionally, the following covariates were tested: current speed (m s^−1^), sea surface temperature (SST, °C), day of year, hour of day, individual tag extent (0 at time of release, 1 at time of departure) and distance to the closest reef (m). The environmental covariates (current speed and SST) were recorded at a weather station 17.7 km from the study area (electronic supplementary material, figure S3). The different models were ranked and selected based on model Akaike information criterion (AIC).

## 3. Results

### 3.1. Residency and site fidelity

**Figure 2.**
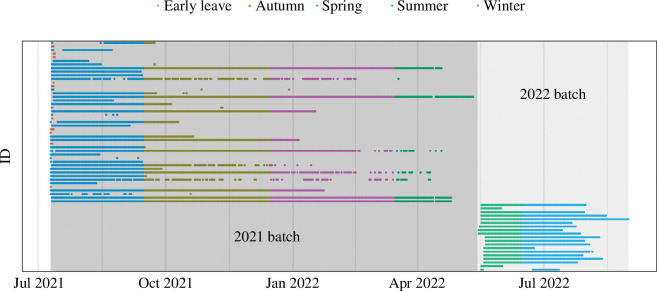
Overview of tag detections over the entire monitoring period. Colours represent the seasonal timing and the labelling of individuals who left the monitoring area prematurely (i.e. residency below 5 days).

In 2021, 14 individuals left the study area within 5 days after release (figure 2 and electronic supplementary material, table S1), while in 2022, all individuals were residents in the study area for at least 15 days. Overall, residency between the two tagging batches differed significantly (figure 2 and electronic supplementary material, figure S4*a*). Mean residency was 97.6 days for the 2021 batch and 65.5 days for the 2022 batch (table 2). Notably, for the 2021 batch, residency greater than 280 days was observed for only three fish. This cut-off is most likely associated with the estimated battery life of 278 days of the tags, meaning true residency of those individual fish may be longer. In contrast, maximum residency for the 2022 batch was 107 days. Most fish left the area at the end of September 2021 and at the end of August 2022 (electronic supplementary material, figure S4*b*).

**Table 2.** Fidelity, home range and residency across different locations. *N* is the number of home-range polygons over which metrics are computed. Mean and s.d. for fidelity and home-range metrics are computed across all days and all individuals. Mean and s.d. for residency are computed across all individuals. In 2022, no individual visited the turbine (L08).

	location	home-range mean (m^2^)	home-range s.d. (m^2^)	fidelity mean	fidelity s.d.	*N* days mean (no.)	*N* days s.d. (no.)	*N* (no.)
2021	all	1129	492	0.93	0.14	97.6	87.2	2632
	other	1894	1691	0.44	0.36	3.9	4.4	77
	L08	2593	1294	0.66	0.13	1.0	–	2
	R01	1042	334	0.94	0.09	65.8	94.7	855
	R02	1017	353	0.94	0.07	18.6	26.6	130
	R03	1225	371	0.94	0.08	62.0	47.3	558
	R04	1102	405	0.94	0.08	59.4	71.1	1010
2022	all	1439	571	0.86	0.18	65.5	25.2	928
	other	1484	861	0.52	0.36	4.5	4.0	68
	L08	–	–	–	–	–	–	
	R01	1317	492	0.86	0.14	18.0	25.4	126
	R02	1323	477	0.90	0.09	55.4	33.1	443
	R03	1879	637	0.72	0.20	16.0	16.6	48
	R04	1615	569	0.91	0.10	30.4	33.4	243

**Figure 3.**
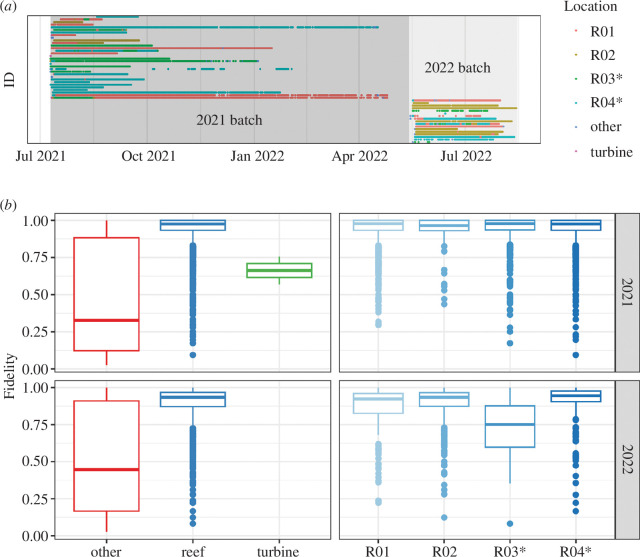
Site visiting and site fidelity. (*a*) Detection of tagged fish and association with different sites. (*b*) Site fidelity across the various structures in the monitoring area. The category ‘other’ includes structures other than the artificial reefs and the turbine (e.g. acoustic receivers or unidentified features). Scour protection was installed around the monopile and the southernmost artificial reefs (R03–R04, labelled with an asterisk).

Cod resided at the four artificial reefs for periods of time between 31.3 and 93.6 days on average in 2021 and between 16.7 and 59 days on average in 2022 (table 2). Moreover, only three individuals resided at a single site less than 80% of their time and other individuals spent most time at a single reef (figure 3*a*, electronic supplementary material, table S2). The monopile was only marginally visited by three individuals of the 2021 batch over 1 day each around the time of release. In few instances, 16 fish stayed for limited time periods at locations not associated with one of the artificial reefs or the turbine (e.g. acoustic receivers or unidentified features, electronic supplementary material, table S2). This occurred mainly just after release or just before the animal left the monitoring area. The home-range size varied between reefs and was larger for the 2022 batch (table 2).

Site fidelity between the artificial reefs, the turbine and other structures differed (figure 3 *b*, left panels, table 2). Fidelity was close to 1 at the reefs, meaning cod stayed consistently at these locations all day, in contrast to other locations (table 2). Across all tagged fish, the marginal visit to the turbine limited the comparison. Fidelity for the southern reefs (R03–R04) with scour protection and the northern reefs (R01–R02) without scour protection were of similar magnitude (figure 3 *b*, right panels), except for a lower fidelity for R03 in 2022, owing to receiver losses around this reef in 2022, which impacted the effective TDOA positioning and induced large positioning error (electronic supplementary material, figure S5).

### 3.2. Inferring hiding in artificial structures

**Figure 4.**
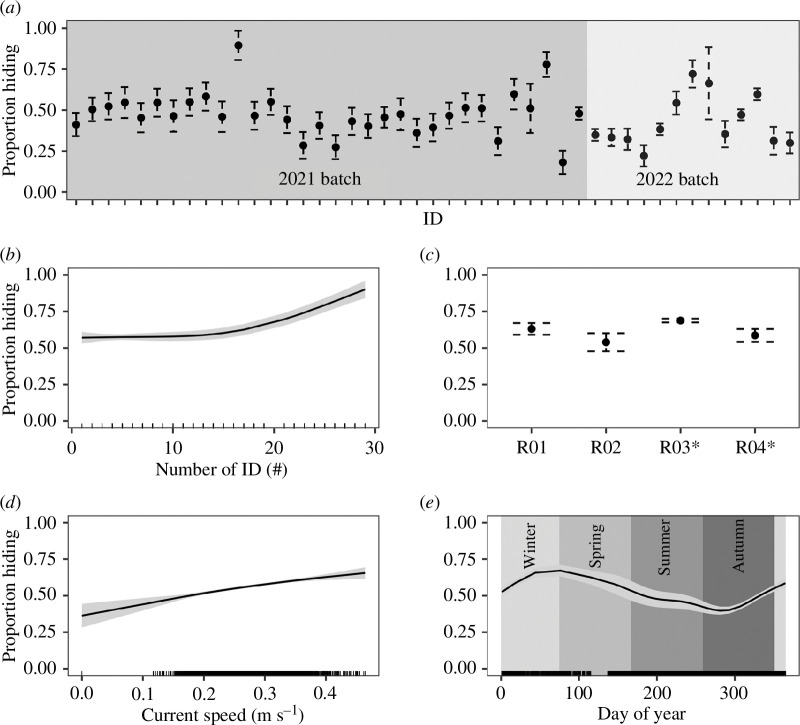
Results of the GAM model for the daily proportion of hiding in the artificial reefs. (*a*) Proportion of hiding for each individual fish. (*b*) Proportion of hiding in relation with daily number of individual ID present (i.e. saturation of acoustic network). (*c*) Proportion of hiding at the different reefs. Scour protection was installed around the monopile and the southernmost artificial reefs (R03–R04, labelled with an asterisk). (*d*) Proportion of hiding as a function of current speed. (*e*) Proportion of hiding as a function of day of year.

The HMM dedicated to inferring hiding in the concrete structures yielded four distinct different states (HS1–HS4) across the different data streams (electronic supplementary material, figure S6 and table S3). The further generalized additive model (GAM) modelling of the daily proportion of the hiding state (HS1) allows us to investigate the effect of different variables individually. Hiding proportion varied between individuals (figure 4*a*) with mean values ranging from 0.18 to 0.89. Differences in the hiding proportion between reefs were limited (figure 4*b*). In contrast, current speed, daily number of individual ID detected (i.e. acoustic network load) and day of year exemplify strong relationships. First, with increased current speed, hiding of the animals in the artificial structures is increased (figure 4*c*). Second, the relationship with the number of animals in the monitoring area (figure 4*d*) shows an increase in hiding proportion when approximately 15 or more daily individual animals are detected. The proportion of hiding is stable when under approximately 15. This might be caused by the increased collision risk and subsequent signal loss when more than 15 individuals are present. The use of GAM modelling allows us to isolate this effect to investigate other variables independently. Last, contrasting trends can be observed at different times of year (figure 4*e*), with the lowest point reached at the end of summer and the peak in hiding reached in winter.

### 3.3. Behavioural states and spatial distribution

**Figure 5.**
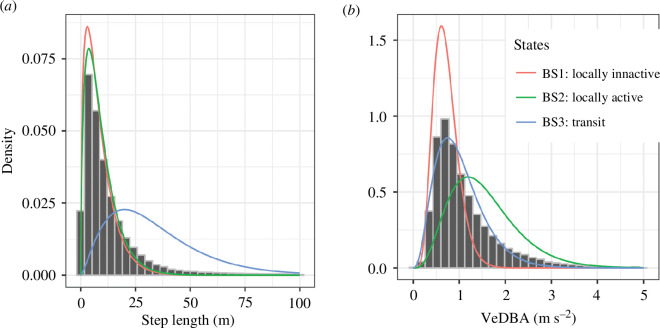
Definition of HMM behavioural states. The data streams used for the model are step length (*a*) and dynamic body acceleration (*b*). Both data streams were modelled using a Gamma distribution, parametrized with mean and s.d.

**Figure 6.**
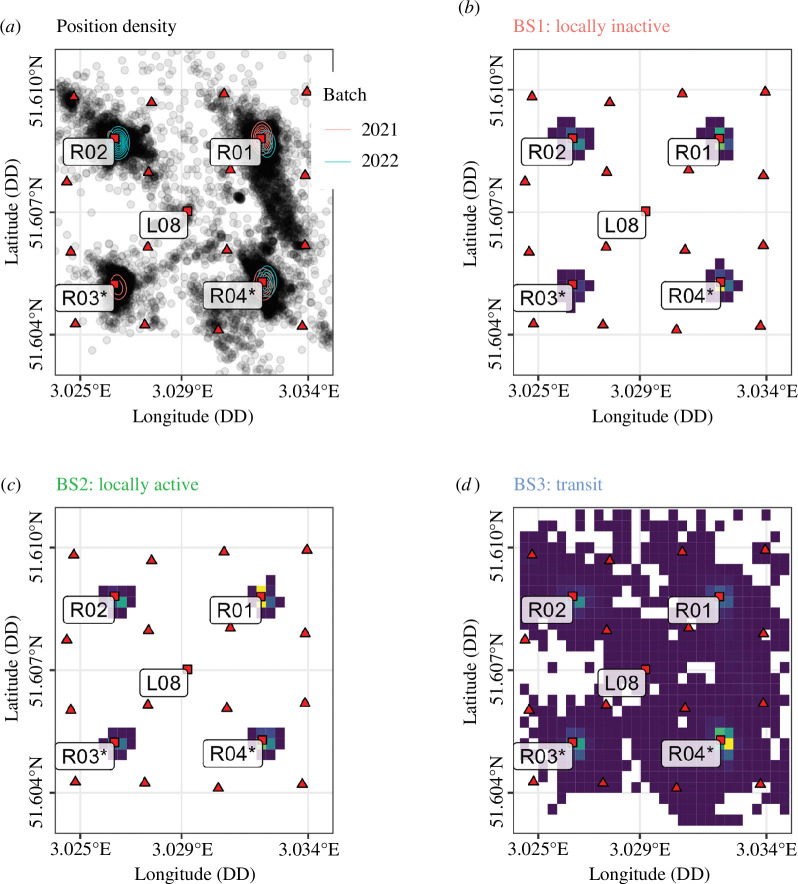
Spatial distribution of positions and states. (*a*) Spatial distribution of positions inferred from acoustic telemetry. The associated densities for the 2021 and 2022 batches are estimated using a two-dimensional kernel density estimation. The positions are concentrated around the artificial reefs (R01–R04), as shown by the inferred density distributions. (*b*) Spatial distribution of the locally inactive state (BS1). (*c*) Spatial distribution of the locally active state (BS2). (*d*) Spatial distribution of the transit state (BS3). Scour protection was installed around the monopile and the southernmost artificial reefs (R03–R04, labelled with an asterisk).

A spatial HMM was used to capture the dynamics in animal behaviour. The optimal covariate configuration was found as ID, SST, current speed, day of year, hour of day, individual tag extent and distance to closest reef (m). The optimal model (figure 5, electronic supplementary material, figures S8, and tables S4 and S5) identified the three BSs as follows: (BS1) low mean and s.d. for both step length and VeDBA; (BS2) low mean and s.d. for step length and large mean and s.d. for VeDBA; (BS3) high mean and s.d. for step length and low mean and high s.d. for VeDBA. The spatial distribution of the TDOA positions was highly concentrated around the artificial reefs (figure 6*a*). The concentration of the local BSs (BS1 and BS2) was concentrated around the artificial reefs (figure 6*b,c*) while the transit state (BS3) was more widespread (figure 6*d*).

## 4. Discussion

### 4.1. Attractiveness of the tested artificial reef types

This study demonstrated that cod were strongly attracted to the two types of artificial reefs with and without scour bed. This was exemplified by the high residency and fidelity at the reefs, but also local behaviours (i.e. locally inactive and active) as well as hiding behaviour, being concentrated around these structures. The residence time varied between the two monitoring periods, with ranges comparable to other studies [9,12,17,19]. The values reported here for fidelity are particularly high (ranging 0.8–1, table 2), e.g. in comparison with Reubens *et al*. [19], which reports fidelity ranging 0.13–0.96 with a mean value of 0.7. With the increasing number of telemetry studies on cod in OWFs, future meta-analyses on generic behavioural patterns in relation to environmental conditions and hard substrate features and comparison between different OWFs would be recommended to assess significance.

There were no clear differences in attractiveness between the reefs with scour protection (R03 and R04) compared with the reefs without (R01 and R02). In addition, even though no cod could be caught around the turbine (L08), this location is marginally visited by all individuals tagged. In this specific set-up, the pipe structures of the artificial reefs provided higher attraction compared with the turbine. Moreover, the artificial reefs with scour protection did not yield higher attraction compared with those without scour protection. The attractivity of hard substrate has been shown for hard-bottom species [6] and the seemingly higher attractiveness for the concrete pipe structures compared with the turbine scour protection bed might be related to the presence of more shelter in the pipes. The modelling of hiding behaviour in the concrete pipes reveals a high daily proportion of hiding (figure 4*a*), i.e. strong usage of the features of the artificial structures. The comparable site fidelity and residency between the two reef types potentially reveal the lower importance of scour protection compared with the presence and type of reefs.

It is known that artificial reefs are attractive to a wide range of demersal fish species [5,19] but the level of attraction can vary between different designs and scour protection [6,24]. A comparative study along the North-American Atlantic coast on five different types of reefs, i.e. natural rocky reefs, concrete pipes, concrete reef balls, concrete Atlantic pods and metal shipwrecks [41] showed similar reef complexity, expressed as digital reef rugosity (DRR) [42], and supported similar fish abundance, biomass and community composition for the three concrete artificial reefs when compared with natural rocky reefs. Metal shipwrecks even had a higher DRR and supported different fish communities than the three concrete artificial reef types and natural rocky reefs. Thus, the more complex the habitat, i.e. the reef structure, the more attractive [43,44]. Lemoine *et al*. [41] recommend concrete pipe-type artificial reefs should be deployed if the objective is to mimic rocky reefs, whereas deploying shipwrecks may create habitats that surpass natural reefs in fish abundance and biomass but with different communities. At present, detailed telemetric studies for Atlantic cod determining local residency and fidelity to different types of artificial reefs and compared with shipwrecks are not available. One study in a Belgian OWF showed that tagged cod that were caught at monopiles were also attracted to nearby reef balls [32].

### 4.2. Differences between the two study years

Differences in site residency, fidelity and timing of leaving the study area were found for the two batches of cod tagged in 2021 and 2022. Cod from the 2022 batch had lower residency (figure 2) and larger home range (table 2) and left the study area earlier in the year, than the 2021 batch. This confirms that cod has a strong tendency to reside in the area in summertime and especially around the artificial structures [12] with the possibility for some individuals to remain in the same OWF throughout the year [9]. The difference in residency could be owing to (i) the timing of tagging, May in 2021 compared with July in 2022, (ii) the temperature profiles, with higher temperatures occurring during 2022 (electronic supplementary material, figure S3; above 21°C compared with 19°C in 2021), or (iii) unidentified disturbing activities in the vicinity of the site. Tagging earlier in the year may concern younger cod, which was reflected in the catch length distribution (electronic supplementary material, figure S1), for which perhaps a higher fraction of the individuals had not settled yet when transiting from wintering to summer grounds, than when caught later in the summer season [12]. Within Atlantic cod, southern North Sea cod show the highest temperature tolerance; however, an upper temperature limit has not been identified yet [45]. The current datasets are too limited to disentangle these potential timing and temperature effects underlying year-to-year differences as well as any potential impact from anthropogenic activities, but the dataset can be used in future meta-analyses comprising more studies.

### 4.3. Biological interpretation of behaviour states

The spatial HMM yielded three different behavioural types/clusters (BS1−BS3). These states are consistent with other studies on Atlantic cod [17,40]. Because no direct observation of their behaviours in a direct ecological context is available, it is difficult to link these states directly to underlying behaviour. Given the length distribution, the smaller individuals were still juveniles while the largest individuals might concern first spawners [46]. BS1 might be more related to sheltering behaviour, whereas BS2 might be more related to actively seeking and handling prey, i.e. feeding behaviour, and possibly also BS3 might include feeding behaviour or excursions outside the artificial reefs. Given that a large proportion of their time budget is spent in the direct vicinity of the artificial reefs, it is likely that these play an important role as feeding habitat as well as sheltering. The sheltering function of the reefs is furthermore demonstrated through the inferred hiding behaviour of cod in the concrete pipes, based on the non-spatial HMMs in relation to current speed and seasons (figure 4*d,e*). This is in line with evidence of local production in cod at artificial reefs in the southern North Sea as found in other OWF studies [12,21]. To further address behaviour of cod in relation to the role and functioning of these newly created reefs, a combination of different techniques and methods is needed, e.g. three-dimensional telemetry, passive and active acoustics, visual observations/cameras, ichthyoplankton, genetics, diet and stable isotope studies.

### 4.4. A broader perspective and implications

In order to quantify the consequences of the findings presented here on population levels, for instance in relation to the ‘attraction–production issue’ [13,14], additional data next to the behavioural tracking data need to be acquired. First, the hereby dataset comprises only a limited area containing four artificial reefs and one monopile, and no larger-scale movement could be tracked. Monitoring of migration routes, migration success and link with artificial reefs at large would be essential to establish any positive net effect on population levels. The rapid developments towards a large European network of acoustic telemetry arrays can make this possible [47]. Second, more insight into the role and functioning of the reefs in relation to feeding and energy expenditure can be gained, e.g. by using diet and stable isotope studies in combination with dynamic energy budget (DEB) modelling [48,49]. Finally, local cod abundance and biomass of aggregations near the reefs can be determined, e.g. by active or passive acoustics [50] and in combination with sampling surveys, in relation to overall population-level assessments and population structure in the southern North Sea [51].

The results presented show a strong attraction to the artificial pipe structures with or without scour bed and indicate that adding more shelter to artificial reefs, thus making them more complex, makes them highly attractive for cod. An explanation for this attraction to the pipe structures is most likely that they mimic natural hard substrate better than scour bed alone, i.e. oyster reefs would have also provided large hiding places. This result is in line with results from a meta-analysis study done by Paxton *et al* [52] that analysed studies comparing fish communities at natural and artificial reefs. They found that across reef ecosystems, artificial reefs support comparable levels of fish density, biomass, species richness and diversity to natural reefs, but that materials choice in artificial reefs is important [41,52]. Thus, the proper design of artificial reefs is important for species attraction and protection [24]. The types of artificial reefs could aid in the creation of multi-purpose areas (North Sea 2016–2021 Policy Memorandum, https://www.government.nl/binaries/government/documenten/policy-notes/2015/12/15/policy-document-on-the-north-sea-2016-2021/nz-eng-beeldscherm.pdf) inside OWFs in which ecological goals (EU Biodiversity Strategy for 2030, https://environment.ec.europa.eu/strategy/biodiversity-strategy-2030_en) are met by creating artificial biogenic temperate reef structures lost to the North Sea.

## 5. Conclusion

This study provides insights into the use of two types of artificial reef structures (concrete pipes of different sizes with and without a scour bed) by Atlantic cod in an OWF. Results from a fine-scale acoustic telemetry study demonstrated that these artificial reefs attracted cod, which showed high fidelity and residency at both types of artificial reefs. Modelling of cod behaviour also revealed different types of local behaviours around the reefs and a substantial proportion of their time hiding in the structures. Artificial reefs that included scour bed protection did not exemplify a significant additional effect of this hard-bottom structure in relation to their attractiveness for the cod. It has been shown that rock scour protection and turbines are highly attractive for cod [6,19,24], but our results suggest that the artificial reefs investigated in this study potentially provide more attractive features, such as the ability to hide in the structures for protection (e.g. against high current speeds). If the current lack of areas with hard substrate habitat in the southern North Sea is a true constraint in the ontogenetic population development of cod, then adding artificial reefs consisting of concrete pipes can serve as a suitable, low-cost and efficient measure to enlarge this area. This can be done in addition to the relatively limited area that the scour bed structures around turbines provide in current and planned OWFs in the North Sea.

## Data Availability

Data and supporting scripts are freely available for on Dryad [53]. Supplementary material is available online [54].

## Footnotes

^1^
https://www.innovasea.com/fish-tracking/products/fathom-software/

## Dedication

Dedicated to the memory of O. van Keeken (1975–2024).

## References

[1] Airoldi L , Balata D , Beck MW . 2008 The gray zone: relationships between habitat loss and marine diversity and their applications in conservation. J. Exp. Mar. Biol. Ecol. **366** , 8–15. (10.1016/j.jembe.2008.07.034)

[2] Olsen TO . 1883 The piscatorial atlas of the North Sea, English Channel, and St. George’s Channels: illustrating the fishing ports, boats, gear, species of fish (how, where, and when caught), and other information concerning fish and fisheries. London, UK: Taylor and Francis.

[3] Airoldi L , Beck MW . 2007 Loss, status and trends for coastal marine habitats of Europe. In Oceanography and marine biology: an annual review (eds RN Gibson , RJA Atkinson , JDM Gordon ), pp. 345–405, vol. **45** . Boca Raton, FL: CRC Press. (10.1201/9781420050943)

[4] Chirosca AM , Rusu L , Bleoju A . 2022 Study on wind farms in the North Sea area. Energ. Rep. **8** , 162–168. (10.1016/j.egyr.2022.10.244)

[5] Degraer S , Carey DA , Coolen JWP , Hutchison ZL , Kerckhof F , Rumes B , Vanaverbeke J . 2020 Offshore wind farm artificial reefs affect ecosystem structure and functioning: a synthesis. Oceanography (Wash. D.C.) **33** , 48–57. (10.5670/oceanog.2020.405)

[6] Glarou M , Zrust M , Svendsen JC . 2020 Using artificial-reef knowledge to enhance the ecological function of offshore wind turbine foundations: implications for fish abundance and diversity. J. Mar. Sci. Eng. **8** , 332. (10.3390/jmse8050332)

[7] Bergström L , Kautsky L , Malm T , Rosenberg R , Wahlberg M , Åstrand Capetillo N , Wilhelmsson D . 2014 Effects of offshore wind farms on marine wildlife—a generalized impact assessment. Environ. Res. Lett. **9** , 034012. (10.1088/1748-9326/9/3/034012)

[8] Andersson MH , Berggren M , Wilhelmsson D , Öhman MC . 2009 Epibenthic colonization of concrete and steel pilings in a cold-temperate embayment: a field experiment. Helgol. Mar. Res. **63** , 249–260. (10.1007/s10152-009-0156-9)

[9] Winter HV , Aarts GM , van KO . 2010 Residence time and behaviour of sole and Cod in the offshore wind farm Egmond Aan Zee (OWEZ). In Ijmuiden IMARES (report / IMARES Wageningen UR), pp. 1–50, vol. OWEZ_R_265_T1_20100916. See https://edepot.wur.nl/198565.

[10] van Deurs M , Grome T , Kaspersen M , Jensen H , Stenberg C , Sørensen T , Støttrup J , Warnar T , Mosegaard H . 2012 Short- and long-term effects of an offshore wind farm on three species of sandeel and their sand habitat. Mar. Ecol. Prog. Ser. **458** , 169–180. (10.3354/meps09736)

[11] van Hal R , Griffioen AB , van Keeken OA . 2017 Changes in fish communities on a small spatial scale, an effect of increased habitat complexity by an offshore wind farm. Mar. Environ. Res. **126** , 26–36. (10.1016/j.marenvres.2017.01.009) 28231443

[12] Reubens JT , Degraer S , Vincx M . 2014 The ecology of benthopelagic fishes at offshore wind farms: a synthesis of 4 years of research. Hydrobiologia **727** , 121–136. (10.1007/s10750-013-1793-1)

[13] Lindberg WJ . 1997 Can science resolve the attraction–production issue? Fisheries **22** , 10–13. (10.1577/1548-8446-22-4)

[14] Wilson J , Osenberg CW , St. Mary CM , Watson CA , Lindberg WJ . 2001 Artificial reefs, the attraction-production issue, and density dependence in marine ornamental fishes. Aquar. Sci. Conserv. **3** , 95–105. (10.1023/A:1011343312031)

[15] Hallier JP , Gaertner D . 2008 Drifting fish aggregation devices could act as an ecological trap for tropical tuna species. Mar. Ecol. Prog. Ser. **353** , 255–264. (10.3354/meps07180)

[16] Robertson BA , Hutto RL . 2006 A framework for understanding ecological traps and an evaluation of existing evidence. Ecology **87** , 1075–1085. (10.1890/0012-9658(2006)87[1075:affuet]2.0.co;2) 16761584

[17] van der Knaap I , Reubens J , Thomas L , Ainslie MA , Winter HV , Hubert J , Martin B , Slabbekoorn H . 2021 Effects of a seismic survey on movement of free-ranging Atlantic cod. Curr. Biol. **31** , 1555–1562.(10.1016/j.cub.2021.01.050) 33567289

[18] Reubens JT , Braeckman U , Vanaverbeke J , Van Colen C , Degraer S , Vincx M . 2013 Aggregation at windmill artificial reefs: CPUE of Atlantic cod (Gadus morhua) and pouting (Trisopterus luscus) at different habitats in the Belgian part of the North Sea. Fish. Res. **139** , 28–34. (10.1016/j.fishres.2012.10.011)

[19] Reubens JT , Pasotti F , Degraer S , Vincx M . 2013 Residency, site fidelity and habitat use of Atlantic cod (Gadus morhua) at an offshore wind farm using acoustic telemetry. Mar. Environ. Res. **90** , 128–135. (10.1016/j.marenvres.2013.07.001) 23937893

[20] Reubens JT , De Rijcke M , Degraer S , Vincx M . 2014 Diel variation in feeding and movement patterns of juvenile Atlantic cod at offshore wind farms. J. Sea Res. **85** , 214–221. (10.1016/j.seares.2013.05.005)

[21] Mavraki N , Degraer S , Vanaverbeke J . 2021 Offshore wind farms and the attraction–production hypothesis: insights from a combination of stomach content and stable isotope analyses. Hydrobiologia **848** , 1639–1657. (10.1007/s10750-021-04553-6)

[22] Gimpel A , Werner KM , Bockelmann FD , Haslob H , Kloppmann M , Schaber M , Stelzenmüller V . 2023 Ecological effects of offshore wind farms on Atlantic cod (Gadus morhua) in the southern North Sea. Sci. Total Environ. **878** , 162902. (10.1016/j.scitotenv.2023.162902) 36934919

[23] ICES . 2023 Benchmark workshop on northern shelf cod stocks (WKBCOD). ICES Sci. Rep. **5** , 1–425. (10.17895/ices.pub.22591423)

[24] Werner KM , Haslob H , Reichel AF , Gimpel A , Stelzenmüller V . 2024 Offshore wind farm foundations as artificial reefs: the devil is in the detail. Fish. Res. **272** , 106937. (10.1016/j.fishres.2024.106937)

[25] Goossens J , T’Jampens M , Deneudt K , Reubens J . 2020 Mooring scientific instruments on the seabed—design, deployment protocol and performance of a recoverable frame for acoustic receivers. Methods Ecol. Evol. **11** , 974–979. (10.1111/2041-210X.13404)

[26] Smircich MG , Kelly JT . 2014 Extending the 2% rule: the effects of heavy internal tags on stress physiology, swimming performance, and growth in brook trout. Anim. Biotelem. **2** , 16. (10.1186/2050-3385-2-16)

[27] Morris CJ , Green JM . 2002 Biological characteristics of a resident population of Atlantic cod (Gadus morhua L.) in southern Labrador. ICES J. Mar. Sci. **59** , 666–678. (10.1006/jmsc.2002.1228)

[28] Lindholm J , Auster PJ , Knight A . 2007 Site fidelity and movement of adult Atlantic cod Gadus morhua at deep boulder reefs in the western Gulf of Maine, USA. Mar. Ecol. Prog. Ser. **342** , 239–247. (10.3354/meps342239)

[29] Smith F . 2013 Understanding HPE in the VEMCO positioning system (VPS). VEMCO technical report. DOC-005457-01.

[30] Lennox RJ *et al* . 2023 Positioning aquatic animals with acoustic transmitters. Methods Ecol. Evol. **14** , 2514–2530. (10.1111/2041-210X.14191)

[31] Roy R , Beguin J , Argillier C , Tissot L , Smith F , Smedbol S , De-Oliveira E . 2014 Testing the VEMCO positioning system: spatial distribution of the probability of location and the positioning error in a reservoir. Anim. Biotelem. **2** , 1. (10.1186/2050-3385-2-1)

[32] van der Knaap I , Slabbekoorn H , Winter HV , Moens T , Reubens J . 2021 Evaluating receiver contributions to acoustic positional telemetry: a case study on Atlantic cod around wind turbines in the North Sea. Anim. Biotelem. **9** , 1–12. (10.1186/s40317-021-00238-y)

[33] Calenge C . 2006 The package “Adehabitat” for the R software: a tool for the analysis of space and habitat use by animals. Ecol. Modell. **197** , 516–519. (10.1016/j.ecolmodel.2006.03.017)

[34] Skerritt DJ , Robertson PA , Mill AC , Polunin NVC , Fitzsimmons C . 2015 Fine-scale movement, activity patterns and home-ranges of European lobster Homarus gammarus. Mar. Ecol. Prog. Ser. **536** , 203–219. (10.3354/meps11374)

[35] Seaman DE , Powell RA . 1996 An evaluation of the accuracy of kernel density estimators for home range analysis. Ecology **77** , 2075–2085. (10.2307/2265701)

[36] Worton BJ . 1995 Using Monte Carlo simulation to evaluate kernel-based home range estimators. J. Wildl. Manage. **59** , 794. (10.2307/3801959)

[37] McClintock BT , Michelot T . 2018 momentuHMM: R package for generalized hidden Markov models of animal movement. Methods Ecol. Evol. **9** , 1518–1530. (10.1111/2041-210X.12995)

[38] McClintock BT , Langrock R , Gimenez O , Cam E , Borchers DL , Glennie R , Patterson TA . 2020 Uncovering ecological state dynamics with hidden Markov models. Ecol. Lett. **23** , 1878–1903. (10.1111/ele.13610) 33073921 PMC7702077

[39] Wood SN . 2011 Fast stable restricted maximum likelihood and marginal likelihood estimation of semiparametric generalized linear models. J. R. Stat. Soc. Ser. B **73** , 3–36. (10.1111/j.1467-9868.2010.00749.x)

[40] Hubert J , Campbell JA , Slabbekoorn H . 2020 Effects of seismic airgun playbacks on swimming patterns and behavioural states of Atlantic cod in a net pen. Mar. Pollut. Bull. **160** , 111680. (10.1016/j.marpolbul.2020.111680) 33181953

[41] Lemoine HR , Paxton AB , Anisfeld SC , Rosemond RC , Peterson CH . 2019 Selecting the optimal artificial reefs to achieve fish habitat enhancement goals. Biol. Conserv. **238** , 108200. (10.1016/j.biocon.2019.108200)

[42] Dustan P , Doherty O , Pardede S . 2013 Digital reef rugosity estimates coral reef habitat complexity. PLoS One **8** , e57386. (10.1371/journal.pone.0057386) 23437380 PMC3578865

[43] Hunter WR , Sayer MDJ . 2009 The comparative effects of habitat complexity on faunal assemblages of northern temperate artificial and natural reefs. ICES J. Mar. Sci. **66** , 691–698. (10.1093/icesjms/fsp058)

[44] Sherman RL , Gilliam DS , Spieler RE . 2002 Artificial reef design: void space, complexity, and attractants. ICES J. Mar. Sci. **59** , S196–S200. (10.1006/jmsc.2001.1163)

[45] Neat F , Righton D . 2007 Warm water occupancy by North Sea Cod. Proc. R. Soc. B **274** , 789–798. (10.1098/rspb.2006.0212) PMC2093972 17251093

[46] Marty L , Rochet MJ , Ernande B . 2014 Temporal trends in age and size at maturation of four North Sea Gadid species: cod, haddock, whiting and Norway pout. Mar. Ecol. Prog. Ser. **497** , 179–197. (10.3354/meps10580)

[47] Reubens J , Verhelst P , van der Knaap I , Wydooghe B , Milotic T , Deneudt K , Hernandez F , Pauwels I . 2019 The need for aquatic tracking networks: the permanent Belgian acoustic receiver network. Anim. Biotelem. **7** , 1–6. (10.1186/s40317-019-0164-8)

[48] Thomas Y , Flye-Sainte-Marie J , Chabot D , Aguirre-Velarde A , Marques GM , Pecquerie L . 2019 Effects of hypoxia on metabolic functions in marine organisms: observed patterns and modelling assumptions within the context of dynamic energy budget (DEB) theory. J. Sea Res. **143** , 231–242. (10.1016/j.seares.2018.05.001)

[49] Slabbekoorn H *et al* . 2019 Population-level consequences of seismic surveys on fishes: an Interdisciplinary challenge. Fish Fish. (Oxf). **20** , 653–685. (10.1111/faf.12367)

[50] Caiger P , Dean M , DeAngelis A , Hatch L , Rice A , Stanley J , Tholke C , Zemeckis D , Van Parijs S . 2020 A decade of monitoring Atlantic cod Gadus morhua spawning aggregations in Massachusetts Bay using passive acoustics. Mar. Ecol. Prog. Ser. **635** , 89–103. (10.3354/meps13219)

[51] ICES . 2020 Workshop on stock identification of North Sea Cod (Wknscodid). ICES Sci. Rep. **2** , 1–82. (10.17895/ICES.PUB.7499)

[52] Paxton AB , Shertzer KW , Bacheler NM , Kellison GT , Riley KL , Taylor JC . 2020 Meta-analysis reveals artificial reefs can be effective tools for fish community enhancement but are not one-size-fits-all. Front. Mar. Sci. **7** , 534479. (10.3389/fmars.2020.00282)

[53] Berges B . 2024 Atlantic Cod around artificial reefs in an OWF [Dataset]. Dryad. (10.5061/dryad.j3tx95xnm)

[54] Berges BJP , Knaap I van der , Keeken O van , Reubens J , Winter HV . 2024 Supplementary material from: Strong site fidelity, Residency, and local behaviour of Atlantic Cod (Gadus Morhua) at two types of artificial reefs in an offshore wind farm. Figshare. (10.6084/m9.figshare.c.7317216) PMC11285481 39076370

